# Effect of the COVID-19 Pandemic on Consumers’ Impulse Buying: The Moderating Role of Moderate Thinking

**DOI:** 10.3390/ijerph182111116

**Published:** 2021-10-22

**Authors:** Shuyang Wang, Yun Liu, Yingying Du, Xingyuan Wang

**Affiliations:** School of Management, Shandong University, Jinan 250100, China; wangshuyang@sdu.edu.cn (S.W.); 202120491@mail.sdu.edu.cn (Y.D.); wangxingyuan@sdu.edu.cn (X.W.)

**Keywords:** COVID-19, impulse buying, sense of control, anxiety, moderate thinking

## Abstract

Based on event systems theory, this study examined the impact of the COVID-19 pandemic on consumers’ impulse buying, as well as the underlying mechanisms and boundary conditions from the perspective of individual consumers. Results of three experiments (N = 437) show that, first, the COVID-19 pandemic enhanced consumers’ impulse buying behavior. Second, two key elements, loss of control and anxiety, mediated the relationship between the COVID-19 pandemic and impulse buying; and third, moderate thinking (also known as Zhong-Yong thinking) moderated the relationship between the COVID-19 pandemic and impulse buying. The findings indicate that in consumers with low moderate thinking, the COVID-19 pandemic has had a stronger effect on impulse buying and has mediated more between the loss of control and anxiety. Conversely, in consumers with high moderate thinking, COVID-19 has had a weaker effect on impulse buying and has mediated less between loss of control and anxiety. This study extends the application of event systems theory and enriches the literature on how the COVID-19 pandemic affects consumer behavior. Furthermore, it provides strategic recommendations for government and consumer responses to COVID-19 pandemic shocks.

## 1. Introduction

The COVID-19 pandemic, a fast-spreading, global public health event, has had a tremendous impact on the global public health system, economy, society, and people’s well-being [1]. With the continuous mutation of the virus, the complete elimination of the COVID-19 virus from the world in a short period of time has been difficult to achieve, and people have had to adapt to a lifestyle under a “normalized epidemic prevention and control” model. Frequent quarantine and social distance control have become the norm to increase the prevention and control of COVID-19 [2].

It has been shown that social isolation caused by the COVID-19 pandemic can lead to lifestyle changes such as sleep disruption, changes in eating habits, and decreased physical activity [3]. The COVID-19 pandemic was found to decrease an individual’s perceived control of self, which in turn caused them to increase their intake of high-calorie, heavy foods [2]. She et al. [4] found that the COVID-19 pandemic caused more stress, depression, and anxiety in individuals. Yiwen and Hahn [5] found that the COVID-19 pandemic reduced employees’ sense of security, which in turn increased moral disengagement and counterproductivity at work. Sun et al. [6] and Wang et al. [7] found that the COVID-19 pandemic triggered uncertainty and awe among consumers, which in turn increased their preference and consumption of green products. Heydari et al. [8] and Asadollahi et al. [9] used qualitative and quantitative methods to find the impact of the COVID-19 epidemic on the sports industry and sports events.

In regions with more severe pandemics, fears of shortages and price increases have triggered large-scale impulse buying, which has disrupted livelihood patterns and social stability [10,11]. For example, in April 2020, Americans spent 18% more on impulse buying, with an average expenditure of USD 182.98 [12]. The British also spent an average of GBP 13 more per person, per week during the COVID-19 pandemic [13]. In China, the scarcity of medical protective supplies also significantly increased consumers’ impulse buying [11]. Impulse buying was a common occurrence during the COVID-19 pandemic, which not only disrupted public order and was detrimental to the macro-control of the pandemic, but also disrupted people’s rhythm of life and compromised their health.

Reviewing the literature, existing studies have discussed consumer behavior in the context of the COVID-19 pandemic to some extent. However, these studies have often taken the COVID-19 pandemic as the research context, ignoring the direct impact and mechanism of action of the COVID-19 pandemic on consumer impulse buying, and there have been theoretical gaps. This study attempts to directly construct the relationship between the COVID-19 pandemic and consumer impulse buying and investigate the intrinsic mechanism and external boundary conditions of this relationship. Based on this, the following research questions are proposed to advance the theory and research on the impact of the COVID-19 pandemic at the consumer level: (1) Does the COVID-19 pandemic increase impulse buying? (2) What psychological mechanisms exist in the relationship between the COVID-19 pandemic and impulse buying? (3) What are the boundary conditions in the relationship between the COVID-19 pandemic and impulse buying?

To answer the research questions in this study, we developed a moderated mediation model to explore the impact of the COVID-19 pandemic on impulse buying and tested the relevant hypotheses through three experiments. Specifically, the COVID-19 pandemic as an external threat has caused consumers more anxiety, stress, and a reduced sense of control [14,15]. For this reason, they have needed a way to cope with external threats [16], such as impulse buying. In addition, specific cultural values and ideological systems affect consumers’ emotional responses to threats and the way they respond [17], which is a key boundary condition for this paper’s model. Specifically, we examined the influence of the unique cultural temperament of moderate thinking (also known as Zhong-Yong thinking). Event systems theory and its related research suggest that individuals subjectively encode, interpret, and make differential interpretations of their environment [18,19,20]. Individuals with moderate thinking can adjust their self-behavior according to the environment while emphasizing self-restraint [21,22], and therefore may have mitigated negative emotional and behavioral reactions triggered by the COVID-19 pandemic.

This study made the following theoretical contributions, particularly in understanding consumer responses to the COVID-19 pandemic. First, this study explored the impact of the COVID-19 pandemic on impulse buying based on event systems theory, which enriches the application scenario of event systems theory and extends research related to the COVID-19 pandemic to the consumer level. Second, this study extended the previous findings by further exploring the mechanisms underlying the impact of the COVID-19 pandemic on impulse buying, namely, the mediating role of the sense of control and anxiety. Third, this study introduced the specific cultural value of moderate thinking into consumer research from the perspective of individual differences, expanding the application of moderate thinking in consumer research.

## 2. Literature Review and Hypotheses

### 2.1. Impulse Buying

As a common behavior in everyday life, impulse buying refers to the unplanned purchase behavior of individuals under the influence of external stimuli [23]. Impulse buying usually has the following three characteristics: (1) it is an unpredictable, sudden behavior that usually occurs simultaneously with an impulse; (2) it is relatively short in duration; and (3) consumers develop the intention to make an impulse purchase but may not do so because of external forces that constrain them. Impulse buying is manifested by consumers purchasing unplanned goods [24] and abandoning the optimal choice for the situation at hand [25].

The factors influencing impulse buying are divided into two main categories: endogenous and exogenous [26]. In terms of endogenous factors, consumer traits, motivation, emotions, gender, and age influence impulse buying [27]. Specifically, individual impulsive tendencies (traits) have a strong positive effect on impulse buying [26,28]. Moreover, sensory seeking (e.g., thrill-seeking, variety-seeking, and curiosity-seeking) can contribute to an individual’s desire to make impulse buys [29,30,31]. Besides, consumer motivations (e.g., hedonic and utilitarian motivations) are important factors that encourage individuals to make impulse buying [24]. Some studies have also highlighted the age and gender differences in impulse buying; for example, Kacen and Lee [32] found that younger consumers have less control over their emotions than older consumers and are therefore more likely to make impulse purchases of products. Dittmar et al. [33] also found that women and men use different patterns of thinking in response to external stimuli and thus make impulse buys for different types of products.

In terms of exogenous factors, store atmosphere, advertising, product appearance, and price can have an impact on impulse buying [34]. For example, some studies have suggested that store music and lighting, as non-verbal communication forms, increase store ambience, which in turn triggers consumers’ desire to make impulse buys [35,36]. Kimiagari and Asadi Malafe [37] identified price as a direct predictor of impulse buying. In addition, Stern [38] found that design is a key factor for product differentiation and positively influences consumer impulse buying.

Related scholars have conducted a large amount of research on the factors influencing impulse buying, which has provided useful insights. However, these studies have mostly focused on general marketing strategies and consumer characteristics and have lacked the exploration of the relationship between public health events and impulse buying, especially the impact of the COVID-19 pandemic.

### 2.2. COVID-19 Pandemic and Impulse Buying

Event system theory states that the stronger the event (the more novel, disruptive, and critical), the more attention and resources it attracts from individuals, which in turn has a greater impact on their behavior [39,40]. The COVID-19 pandemic is characterized by all three of these features. First, the COVID-19 pandemic is novel because it is a new, emergent public health event that lacks clear and established procedures to guide people’s behavior in life and work [19]. Second, the COVID-19 pandemic is devastating; it has had a huge impact on the global economy, society, and people’s well-being [1]. Third, the COVID-19 pandemic is critical because it has severely affected the psychological state of consumers, causing increased stress and decreased security [2,3,4,10]. Therefore, how to change this state is an urgent issue for consumers. Thus, at the consumer level, the COVID-19 pandemic, as a major public event, has had an impact on consumer psychology and behavior.

Specifically, the COVID-19 pandemic has induced impulse buying in consumers. It has changed the environment in which consumers live; for example, it has triggered policy changes (e.g., home isolation, social distancing, and travel restrictions) and health risks (e.g., infection and death) [41,42,43,44,45]. This change has not only provoked consumers’ fear of death but has also brought more negative emotions and uncertainty to consumers [7]. The fear of death can increase consumers’ urge to seek and consume material goods [46]. This is because impulse buying can bring psychological and physiological comfort to consumers, while consciously diverting their attention away from the pain of the threat of death. Similarly, Mandel and Smeesters [47] found that when consumers are faced with the fear of death, they tend to divert their attention through overeating and hedonistic food consumption. Besides, it has been suggested that consumer emotions are an important influence on impulse buying because consumers can use impulse buying to relieve negative emotions [48] and to obtain pleasure [49]. Furthermore, from a neuromarketing perspective, the emotional experiences of anxiety and fear induced by the COVID-19 pandemic all cause significant activation of the amygdala [50], increasing impulsive and risk-taking behaviors (e.g., impulse buying) [51]. Therefore, the following hypothesis is proposed in this study.

**Hypothesis** **1** **(H1).** *The COVID-19 pandemic positively affects consumer impulse buying.*

### 2.3. Mediating Role of Sense of Control and Anxiety

Sense of control is defined as an individual’s beliefs about the extent to which they can influence events to meet expectations [52,53]. Sense of control is usually reflected in two ways: the first is the individual’s control over the external environment, that is, the extent to which the individual believes that the external environment hinders the achievement of goals. The second is the individual’s control over themselves and their behavior, that is, the extent to which the individual believes they are capable of achieving their goals and controlling their environment [54]. In the present study scenario, the COVID-19 pandemic has changed peoples’ lives and work environments [19], for example, through social alienation and travel restrictions. However, the COVID-19 pandemic has also left individuals with uncertainty about whether they are infected with COVID-19 and how to cope with it [7]. Thus, the environmental changes and self-uncertainty brought by the COVID-19 pandemic to consumers have led to a loss of control.

Furthermore, according to the compensatory control theory, individuals have a natural desire to restore their sense of control when it is absent [55,56,57]. Consumers can directly restore their sense of control through self-improvement, but this approach to alleviate the loss of control triggered by the COVID-19 pandemic is more difficult. Consumers can also compensate for their lack of sense of control through external forces. Previous studies have indicated that the lack of control affects individual consumption behavior and consumption patterns, especially impulse buying [58,59]. This is because impulse buying can satisfy the psychological need of individuals to compensate for their sense of control. Therefore, Hypothesis 2 is as follows:

**Hypothesis** **2** **(H2).** *Sense of control mediates the relationship between the COVID-19 pandemic and impulse buying.*

Anxiety is a negative emotional state comprising a complex array of emotions, such as distress, annoyance, and tension due to the uncertainty and unknown nature of events [60,61]. Based on the duration of anxiety, anxiety can be divided into trait anxiety and state anxiety [62]. Trait anxiety has a tendency to be individually variable and stable over time. In contrast, state anxiety refers to a transient emotional experience that is usually triggered by external circumstances, such as panic events and potential threats [63]. The present study focused on exploring state anxiety (hereafter referred to as anxiety) in individuals under the influence of the COVID-19 pandemic. As the most frequently reported problem by individuals during the COVID-19 pandemic, anxiety may be related to the COVID-19 pandemic [64]. Because the COVID-19 virus is highly contagious and potentially severely damaging, and the pandemic is so uncertain, when faced with the potential threat posed by the pandemic, individuals may exhibit anxiety (e.g., worry and stress) about adverse consequences (e.g., illness and death).

As a type of negative emotion, anxiety is a trigger for impulse buying [65]. Individuals can alleviate anxiety through impulse buying as well as revenge buying. For example, Ferraro et al. [46] found that death anxiety increased individuals’ impulses to material purchases as well as consumption. Similarly, Gao et al. [66] noted that anxiety could lead individuals to engage in binge-eating behavior. Therefore, Hypothesis 3 is proposed in this study.

**Hypothesis** **3** **(H3).** *Anxiety mediates the relationship between the COVID-19 pandemic and impulse buying.*

### 2.4. The Moderating Effect of Moderate Thinking

Moderate thinking (also known as Zhong-Yong thinking) refers to “thinking about the same thing from multiple perspectives, and after considering different views in detail, choosing the way that takes into account the self and the big situation” [67]. Moderate thinking emphasizes the need to avoid extremes and seek moderation [68,69,70]. Moderate thinking is not only a Chinese philosophical and cultural value but also a modern Western cultural value [71]. For example, Aristotle believed that a good life requires moderation in behavior, especially between excessive lack and scarcity of resources [72]. In Western culture, moderate thinking can help individuals choose between conflicting goals or attributes [71]. Moderate thinking has three main characteristics: first, plurality, which means thinking from multiple perspectives; second, integration, which means integrating internal and external information; and third, harmony, which means choosing the middle ground and taking the big picture into account. Therefore, when public health events such as the COVID-19 pandemic happen, this kind of systemic thinking that examines the situation and avoids extremes can prevent irrational decisions owing to psychological problems, such as anxiety and a sense of the loss of control.

Event systems theory and its related studies indicate that individuals subjectively encode, interpret, and make differential interpretations of the events they face [18,19,20]. As a specific cultural value, moderate thinking plays an important role in the consumer decision-making process [69,71]. Based on this, we suggest that moderate thinking has played a moderating role between the COVID-19 pandemic and impulse buying. Specifically, individuals with high moderate thinking not only adjust their behavior to the environment when making decisions, but also emphasize self-control and refrain from fluctuating in transient emotions [21,67,68,70]. Thus, in the face of a serious threat posed by the COVID-19 pandemic, consumers with high moderate thinking have made an effective shift from an emotion-based hot processing system to a cognitive-based cold processing system to achieve self-control in their behavior. In contrast, consumers with low moderate thinking have focused only on the COVID-19 pandemic and have allowed negative emotions and feelings to dominate their actions. In contrast, effective emotion regulation can mitigate impulsive behavior to some extent. Based on this, we propose Hypothesis 3.

**Hypothesis** **4** **(H4).** *Moderate thinking moderates the effect of the COVID-19 pandemic on impulse buying. Specifically, the effect of the COVID-19 pandemic on impulse buying is weakened in consumers with high moderate thinking, and the effect of the COVID-19 pandemic on impulse buying is enhanced for consumers with low moderate thinking.*

**Figure 1:**
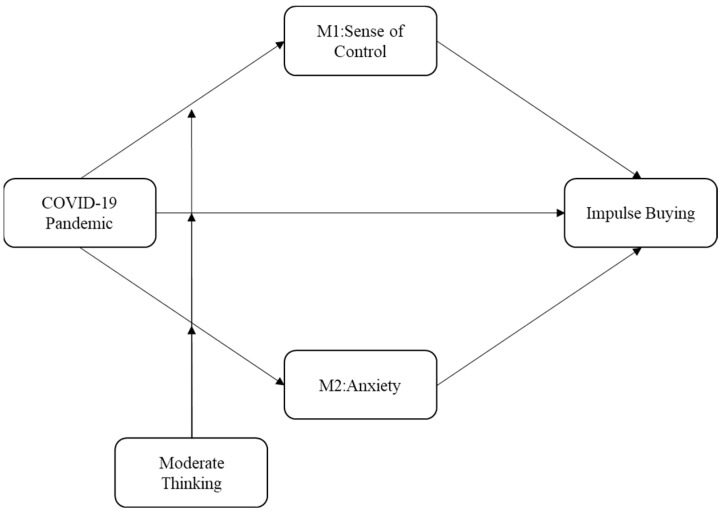
Conceptual model.

In summary, the theoretical model of this paper is shown in Figure 1.

## 3. Overview of Studies

This research used the experimental method, as it is better able to provide evidence of a causal relationship between variables [73]. This paper conducted three independent experiments to explore how the COVID-19 pandemic affects impulse buying, the underlying mechanisms, and their boundary conditions. Study 1 examined the direct effect of the COVID-19 pandemic on impulse buying, that is, H1 was verified. Study 2 enhanced the robustness of Study 1 findings by replacing the material stimuli. In addition, Study 2 further verified the potential mechanisms underlying the effect of the COVID-19 pandemic on impulse buying, that is, H2 and H3 were verified. Study 3 verified the boundary condition for the effect of the COVID-19 epidemic on impulse buying, that is, H4 was verified.

In each of these studies, we reported the materials and methods, results, and discussion. Participants were recruited via Credamo (www.credamo.com) and were paid for their participation. Credamo is a reliable Chinese data collection platform similar to Qualtrics Online Sample [74]. All scales used in the study were adapted or directly used from mature scales. Specifically, The impulse buying was measured with reference to Kukar-Kinne et al. [75] and Ridgway et al. [76]. The sense of control was measured with reference to relevant studies by Lachman and Weaver [77] and Su et al. [78]. Anxiety was measured with reference to Winterich and Haws [79]. Detailed measurement items, reliability and validity are shown in Appendix A and Appendix B. All items were measured on a 7-point Likert scale, with 1 meaning “strongly disagree”, and 7 meaning “strongly agree”.

## 4. Study 1

The purpose of Study 1 was to test our main Hypothesis 1, that is, the effect of the COVID-19 pandemic on consumer impulse buying, using an experimental method. We first manipulated participants’ perceptions of the COVID-19 pandemic through a reading task and then measured participants’ intention to make impulse buys.

### 4.1. Method

First, 120 participants (of which 1 participant failed the attention test and 119 participants were valid) were recruited via Credamo. For this study, 119 participants (53.78% female, *M_age_* = 28.23) were randomly assigned to two groups (COVID-19 pandemic group vs. control group).

The experiment was divided into three parts. First, the procedure of the experiment was briefly explained to the participants, and their consent was obtained. Second, we performed the manipulation of the COVID-19 pandemic and the measurement of consumer impulse buying. In this study, participants were randomly assigned to the COVID-19 pandemic group and the control group. For the manipulation of the COVID-19 pandemic, we referred to the study by Kim et al. [80], where participants in the COVID-19 pandemic group were asked to read a news report about the dangers of the COVID-19 pandemic, while participants in the control group were asked to read a report about a golf article, both of which were similar in length. After reading the news report, participants were asked to recall what they had read as an attention test. Next, subjects were asked to fill out an impulse-buying scale (*α* = 0.93). Finally, demographic variables such as gender and age of the participants were measured.

### 4.2. Results and Discussion

**Figure 2:**
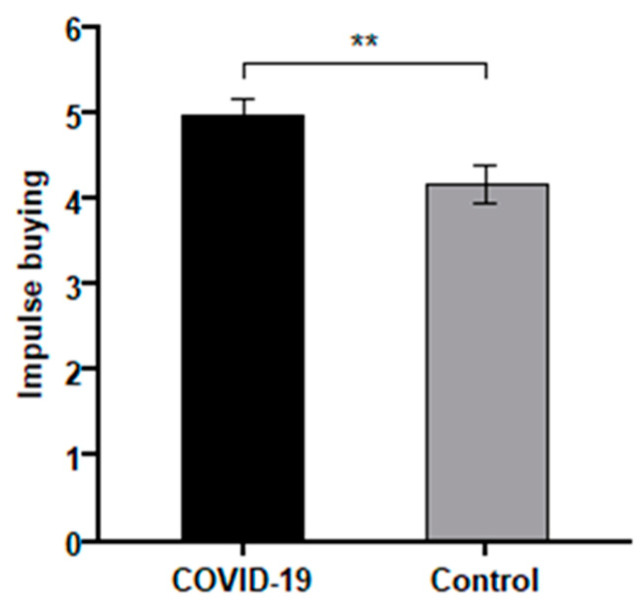
The impact of the COVID-19 pandemic on impulse buying (Study 1). Note: ** *p* < 0.01.

Impulse buying: The one-way ANOVA was conducted with COVID-19 pandemic (COVID-19 vs. control) as the independent variable and impulse buying as the dependent variable. The results show that the COVID-19 pandemic had a significant effect on participants’ impulse buying (*F* (1,117) = 7.11, *p* < 0.01). Specifically, impulse buying scores were significantly higher in the COVID-19 pandemic group (*M _COVID-19_* = 4.97, *SD* = 1.51) than in the control group (*M _control_* = 4.18, *SD* = 1.73, *p* < 0.01). As shown in Figure 2, the COVID-19 pandemic (COVID-19 vs. control) led to higher impulse purchases, and the results supported Hypothesis 1.

Discussion: The results of Study 1 validate the positive effect of the COVID-19 pandemic on consumer impulse buying, consistent with Hypothesis 1. However, Study 1 also had some limitations. We raised some questions for this purpose: first, will the results remain robust if a different group of stimuli and manipulated materials is used? Second, what are the potential mechanisms underlying the effect of the COVID-19 pandemic on consumer impulse buying that have not been verified? To address these questions, Study 2 included other stimulus and manipulation material to enhance the external validity of the experiment, and also to verify further the mediating role of the perception of control and anxiety between the COVID-19 pandemic and consumer impulse buying.

## 5. Study 2

Study 2 had two main purposes. First, Study 2 further validated the robustness of the results in Study 1 by replacing the stimulus material. Second, Study 2 examined Hypothesises 2 and 3, that is, to explore the potential mechanisms underlying the effect of the COVID-19 pandemic on consumer impulse buying, that is, the mediating role of sense of control and anxiety.

### 5.1. Method

First, 120 participants (of which 3 participants failed the attention test, and 117 participants were valid) were recruited via Credamo. For this study, 117 participants (56.41% female, *M_age_* = 31.62) were randomly assigned to two groups (COVID-19 vs. control).

The experiment was divided into three parts. First, the experimental procedure introduction and informed consent statement. Second, we performed the manipulation of the COVID-19 pandemic and the measurement of the variables. In this study, participants were randomly assigned to the COVID-19 pandemic group and the control group, where Study 2 replaced the stimulus material with a different one than Study 1 to verify the robustness of the results. For the manipulation of the COVID-19 pandemic, we referred to the studies of Wang et al. [7] and Koles et al. [81]. Specifically, participants in the COVID-19 pandemic group were asked to read a paragraph of material about the COVID-19 pandemic, while participants in the control group were asked to read material about how a student organized their workspace in preparation for class. After reading the material, participants were asked a simple reading comprehension question as an attention test. Next, participants were asked to fill out scales for sense of control (*α* = 0.94), anxiety (*α* = 0.95), and impulse buying (*α* = 0.90). Finally, demographic variables such as gender and age of the participants were measured.

### 5.2. Results

**Figure 3:**
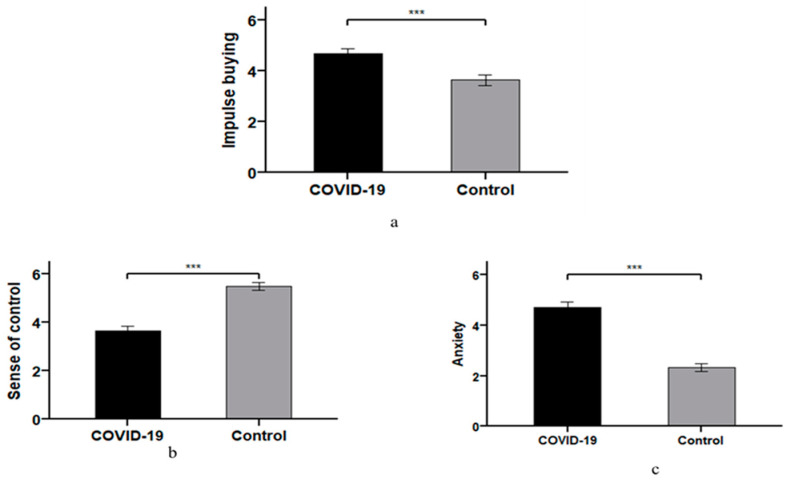
The impact of the COVID-19 pandemic. (**a**) The impact of the COVID-19 pandemic on impulse buying. (**b**) The impact of the COVID-19 pandemic on sense of control. (**c**) The impact of the COVID-19 pandemic on anxiety. Note: *** *p* < 0.001.

Impulse buying: The one-way ANOVA was conducted with the COVID-19 pandemic (COVID-19 vs. control) as the independent variable and impulse buying as the dependent variable. The results show that the COVID-19 pandemic had a significant effect on participants’ impulse buying (*F* (1,115) = 14.64, *p* < 0.001). Specifically, impulse-buying scores in the COVID-19 pandemic group (*M _COVID-19_* = 4.70, *SD* = 1.37) were significantly higher than impulse-buying scores in the control group (*M _control_* = 3.64, *SD* = 1.61, *p* < 0.001). As shown in Figure 3a, the COVID-19 pandemic (COVID-19 vs. control) has led to higher impulse buying, and the results support Hypothesis 1.

Sense of control: The one-way ANOVA was conducted with COVID-19 pandemic (COVID-19 vs. control) as the independent variable and sense of control as the dependent variable. The results show that the COVID-19 pandemic had a significant effect on participants’ sense of control (*F* (1,115) = 55.65, *p* < 0.001). Specifically, the sense-of-control score in the COVID-19 pandemic group (*M _COVID-19_* = 3.67, *SD* = 1.40) was significantly lower than that of the control group (*M _control_* = 5.50, *SD* = 1.25, *p* < 0.001). As shown in Figure 3b, the COVID-19 pandemic (COVID-19 vs. control) has reduced consumers’ sense of control.

Anxiety: A one-way ANOVA was conducted with the COVID-19 pandemic (COVID-19 vs. control) as the independent variable and anxiety as the dependent variable. The results show that the COVID-19 pandemic had a significant effect on participants’ anxiety (*F* (1,115) = 94.68, *p* < 0.001). Specifically, the anxiety scores in the COVID-19 pandemic group (*M _COVID-19_* = 4.72, *SD* = 1.43) were significantly higher than those in the control group (*M _control_* = 2.33, *SD* = 1.22, *p* < 0.001). As shown in Figure 3c, the COVID-19 pandemic (COVID-19 vs. control) has led to higher anxiety.

**Figure 4:**
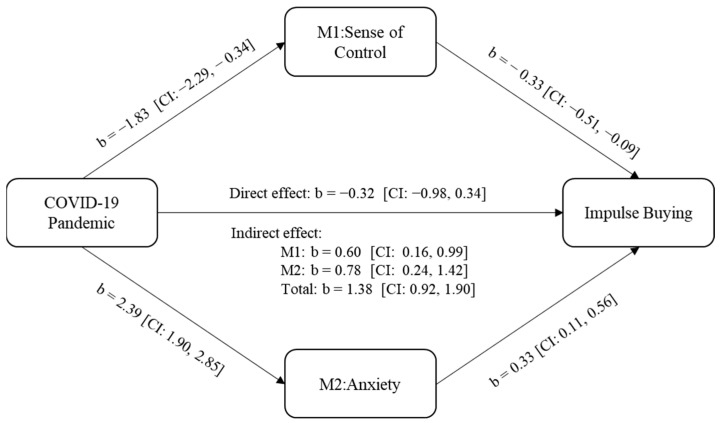
The mediating effect of sense of control and anxiety.

Mediating role of sense of control and anxiety: We examined the mediating role of sense of control and anxiety on the effect of the COVID-19 pandemic on consumer impulse buying using PROCESS 3.5 [82]. The independent variable was the COVID-19 pandemic (control = 0, COVID-19 = 1), the mediating variable was sense of control and anxiety, and the dependent variable was impulse buying. Bootstrapping (sample size = 5000, 95% C.I., model 4) results show that the COVID-19 pandemic had a significant negative effect on sense of control (*b* = −1.83, *p* < 0.001), and sense of control had a significant negative effect on impulse purchase (b = −0.33, *p* < 0.01). As shown in Figure 4, the indirect effect of the COVID-19 pandemic → sense of control → impulse buying was 0.60 (*SE* = 0.21), with a confidence interval excluding 0 (95% *C.I.* = 0.16 to 0.99). The COVID-19 pandemic had a significant positive effect on anxiety (*b* = 2.39, *p* < 0.001), and anxiety on impulse buying (*b* = 0.33, *p* < 0.01). The indirect effect of COVID-19 pandemic → anxiety → impulse buying was 0.78 (*SE* = 0.30), with a confidence interval excluding 0 (95% *C.I.* = 0.24 to 1.42). Therefore, Hypotheses 2 and 3 were supported

### 5.3. Discussion

First, Study 2 again tested Hypothesis 1, the positive effect of the COVID-19 pandemic on consumer impulse buying, by replacing the stimuli. The results of Study 2 support Hypotheses 2 and 3, namely, the mediating role of sense of control and anxiety in the effect of the COVID-19 pandemic on consumer impulse buying. Specifically, compared to the control group, the COVID-19 pandemic decreased consumers’ sense of control and triggered consumers’ anxiety at the same time. In contrast, when consumers’ sense of control was reduced, and anxiety was increased, consumers would be more likely to make an impulse purchase.

## 6. Study 3

The main purpose of Study 3 was to examine the boundary condition of the COVID-19 pandemic on consumer impulse buying, namely, the moderating effect of moderate thinking.

### 6.1. Method

First, 203 participants (of which two participants failed the attention test, and 201 participants were valid) were recruited via Credamo. Study 3 used a mixed method wherein 201 participants (53.73% female, M_age_ = 30.73) were randomly assigned to two groups (COVID-19 vs. control group) in which moderate thinking was the within-group measure.

Study 3 was divided into three parts. First, experimental procedure introduction and informed consent statement. Second, we performed the manipulation of the COVID-19 pandemic with the measurement of variables. The stimulus material for this part was the same as that in Study 2. After reading the material, participants were asked a simple reading comprehension question to serve as an attention test. Next, participants were asked to fill out scales for sense of control (*α* = 0.88), anxiety (*α* = 0.94), impulse buying (*α* = 0.87), and moderate thinking (*α* = 0.95). Finally, demographic variables such as gender and age of the subjects were measured as control variables for the model.

### 6.2. Results

**Figure 5:**
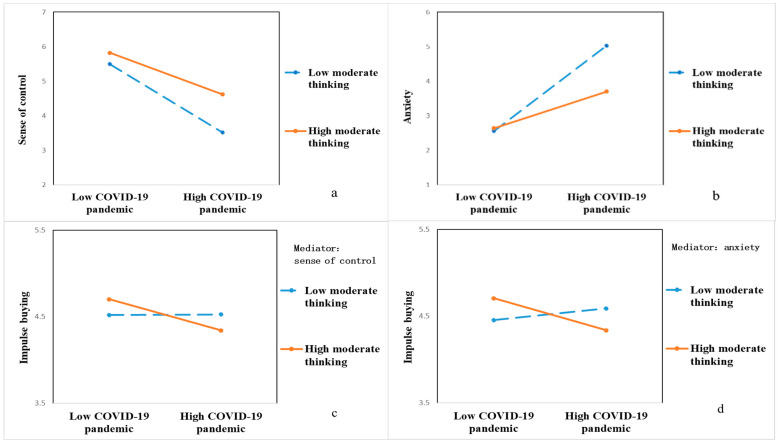
Moderating effects. (**a**) The moderating role of moderate thinking between COVID-19 and sense of control. (**b**) The moderating role of moderate thinking between COVID-19 and anxiety. (**c**) The moderated mediation of sense of control. (**d**) The moderated mediation of anxiety.

We used OLS regression to analyze the data. We first mean-centered the moderate thinking and then tested the moderating effect of moderate thinking on the COVID-19 pandemic. The results of the data analysis are presented in Table 1. Model 1 showed that the COVID-19 pandemic significantly negatively influenced the sense of control (*b* = −1.59, *p* < 0.001), and the interaction between the COVID-19 pandemic and moderate thinking significantly positively influenced the sense of control (*b* = 0.41, *p* < 0.01). Figure 5a depicts the moderating effect of moderate thinking on the relationship between the COVID-19 pandemic and sense of control. Individuals with high moderate thinking were significantly less affected by the COVID-19 pandemic and had a smaller loss of sense of control. Model 2 showed that the COVID-19 pandemic significantly positively affected anxiety (*b* = 1.76, *p* < 0.001), and the interaction between the COVID-19 pandemic and moderate thinking significantly negatively affected anxiety (*b* = −0.74, *p* < 0.001). Figure 5b depicts the moderating effect of moderate thinking on the relationship between the COVID-19 pandemic and anxiety. It can be seen that individuals with high moderate thinking were also less affected by the COVID-19 pandemic, and they felt less anxious. These results suggest that moderate thinking has significantly mitigated and suppressed the adverse effects of the COVID-19 pandemic on consumers’ sense of control and anxiety.

Model 3 showed that the COVID-19 pandemic significantly positively influenced impulse buying (*b* = 0.62, *p* < 0.001). The interaction term between the COVID-19 pandemic and moderate thinking significantly negatively influenced impulse buying (*b* = −0.52, *p* < 0.01); that is, moderate thinking weakened the positive effect of the COVID-19 pandemic on impulse buying. This suggests that the COVID-19 pandemic has not triggered impulse consumption in individuals with a high level of moderate thinking. Thus, Hypothesis 3 is supported. Models 4, 5, and 6 reported the results of the tests with the inclusion of mediating variables. We found that the coefficient and significance of the interaction term between the COVID-19 pandemic and moderate thinking significantly decreased after the inclusion of the sense of control (*b* = −0.33, *p* < 0.05, model 4) and anxiety variables (*b* = −0.26, *p* > 0.1, model 5), which partially supports the moderated mediation effect proposed in this paper.

Moderated mediation. The “conditional indirect effects” test was used to verify the moderating effect of moderate thinking on the mediating effect of sense of control and anxiety. The Bootstrap method was used to conduct a coefficient test with a sample size of 1000 and to calculate the confidence interval for the Bias-Corrected test. In Table 2, the test results for path a show that the indirect effect of COVID-19 pandemic on consumer impulse buying was smaller when the moderate thinking was higher *(b* = −0.36, *p* < 0.01), with an error-corrected confidence interval of [−0.62, −0.18] excluding 0. The test results for path b show that the indirect effect of COVID-19 pandemic on consumer impulse buying was smaller when the moderate thinking was higher (*b* = −0.37, *p* < 0.001), with an error-corrected confidence interval of [−0.58, −0.18] excluding 0. The above results suggest that moderate thinking has moderated the mediating effects of control and anxiety and has significantly inhibited the effect of the COVID-19 pandemic on impulse buying. Figure 5b,c depict the moderating effect of moderate thinking on the indirect effect of the COVID-19 pandemic on impulse buying (path a and path b). It can be seen that the higher the moderate thinking, the smaller the effect of COVID-19 pandemic on impulse buying. Therefore, Hypothesis 4 is well supported.

### 6.3. Discussion

The results of Study 3 support Hypothesis 4, which states that moderate thinking moderates the relationship between the COVID-19 pandemic and consumer purchases. Specifically, moderate thinking weakened the effect of the COVID-19 pandemic on impulse buying. The effect of the COVID-19 pandemic on impulse buying was stronger when consumers had lower moderate thinking. Conversely, the effect of the COVID-19 pandemic on impulse buying was weaker or even absent when consumers had higher moderate thinking. Moreover, Study 3 further tested the moderated mediation effect. Study 3 showed that the mediating effect of sense of control and anxiety was stronger when consumers had lower moderate thinking. Conversely, the mediating effect of sense of control and anxiety was also weaker when consumers had higher moderate thinking.

## 7. General Discussion

### 7.1. Theoretical and Practical Implications

As an unexpected public event at this stage, the COVID-19 pandemic has had a tremendous influence on the global economy, society, and people’s well-being [1], and has become a topic of concern for academics and governments. This study examined the impact of the COVID-19 pandemic on consumers’ impulse buying as well as the potential mechanisms and the boundary conditions based on event systems theory from the individual consumer level. Overall, the main theoretical contributions of this study were as follows.

First, this study examined the impact of the COVID-19 pandemic on individual impulse buying at the consumer level, enriching the field of research on the effects of public health events. Infectious diseases are highly contagious and have serious consequences [83]. Previous studies have explored the economic, market, business, sports industry, and job impacts of the COVID-19 pandemic [8,9,43,84,85,86,87] but have neglected its effects on individual consumer psychology and behavior. This study filled this gap; we found that the COVID-19 pandemic exacerbated consumer impulse buying. This finding not only responded to Das et al.’s [83] call for exploring the relationship between infectious diseases and consumer behavior, but also increased our understanding of the potential links between COVID-19 outbreaks and consumer well-being and consumer behavior, extending the study of the effects of public health events to the consumer field.

Second, this study introduced the event systems theory, a fundamental theory of organizational behavior, to the field of consumer behavior. Event systems theory emphasizes that dynamic events experienced by individuals can have a significant impact on them [39]. Previous research has discussed leader effectiveness [88], dual-career family management [89], employee creativity [18], and career transitions [90] based on event systems theory, but there has been a lack of application of event systems theory to the field of consumer behavior. This study examined the impact of a public health event, the COVID-19 pandemic, on consumer behavior based on event systems theory. Similar to Lin et al.’s [19] findings on the impact of COVID-19 on employee behavior based on event systems theory, this study found that the COVID-19 pandemic disrupted consumers’ psychological states and triggered impulse buying. In conclusion, this study enriched the study of event system theory and expanded its application.

Third, this study clarified the potential psychological mechanisms underlying the impact of the COVID-19 pandemic on impulse buying. Most studies have examined the impact of the COVID-19 pandemic from a macro perspective [41,85]. In contrast, the present study focused not only on the behavioral changes of consumers, but also on the psychological mechanisms behind the behavioral changes triggered by the pandemic. This study found that the COVID-19 pandemic reduced consumers’ sense of control and increased anxiety, which in turn triggered impulse buying. This study provided a psychological perspective for the study of the impact of COVID-19 pandemic events on consumers.

Fourth, this study proposed and tested the moderating role of moderate thinking as a boundary condition in the relationship between the COVID-19 pandemic and consumer impulse buying. The established literature has paid insufficient attention to moderate thinking, especially in the consumer domain. As previously discussed, individuals with high moderate thinking think more holistically [91] and avoid extremes [67,69,71]. Thus, moderate thinking weakens the impact of the COVID-19 pandemic on impulse buying. This study not only responded to Drolet et al.’s [71] call for research on moderate thinking and consumer behavior, but also provided a research basis for understanding the effects of cultural values influencing public health events.

In addition, this study has implications for practice: first, this study found that the COVID-19 pandemic affected consumers’ impulse buying through two pathways: sense of control and anxiety. Although a reduced sense of control and increased anxiety would facilitate consumers’ response to risk, excessive exaggeration would trigger negative effects. Therefore, the government and related organizations should properly guide individuals’ awareness of the COVID-19 pandemic and intervene to reduce their lack of control and anxiety to avoid negative consumer psychology and behavior. For example, the government can mitigate the adverse effects of the COVID-19 pandemic through vaccination. However, the analysis of moderating effects found that moderate thinking would weaken the impact of the COVID-19 pandemic on impulse buying. Therefore, the government can intervene in terms of cultural values to achieve consumer self-regulation and avoid the negative effects of the COVID-19 pandemic. For example, the government and related organizations can help individuals develop the value of moderate thinking so that they know how to take a holistic and dialectical view of the COVID-19 pandemic in their lives.

### 7.2. Research Limitations and Future

There were some shortcomings to this study. First, in the experimental stimuli, we used words and pictures to simulate real scenes; although this ensured that the results had high internal validity, there were some gaps in the real scenes. The findings of this study can be further validated by secondary data and neurophysiological data [92] in the future to increase the external validity of the findings. Second, this study only explored the impact of the COVID-19 pandemic on impulse buying, but the impact of the COVID-19 pandemic on consumer behavior is multifaceted. For example, the COVID-19 pandemic may have affected consumers’ preference for environmentally friendly products or local brands. The impact of the COVID-19 pandemic on other consumer behaviors can be further explored in the future. Third, this study explored the boundary conditions of the impact of the COVID-19 pandemic in terms of cultural values, but there may be other moderating factors. Other boundary conditions, such as Pura Vida thinking (a way of thinking that promotes going with the flow and simply enjoying life), can be further explored in the future.

## 8. Conclusions

Based on event systems theory, this study examined the effect of the COVID-19 pandemic on consumer impulse buying as well as the underlying psychological mechanisms and boundary conditions between the relationships. The findings indicate the following. The COVID-19 pandemic has positively influenced consumers’ impulse buying through the mediating role of sense of control and anxiety. Specifically, the COVID-19 pandemic has changed consumers’ environment and increased their uncertainty, leading to a lack of control and anxiety and ultimately triggering impulse buying. However, the above influence process has been moderated by consumers’ moderate thinking. In particular, the effect of the COVID-19 pandemic on impulse buying has been stronger in consumers with low moderate thinking, and the mediating effect of the sense of control and anxiety has been stronger. Conversely, the effect of COVID-19 on impulse buying has been weaker in consumers with high moderate thinking, and the mediating effect of the sense of control and anxiety has been weaker. This study made some theoretical and practical implications, particularly in understanding consumer responses to the COVID-19 pandemic. First, this study explored the impact of the COVID-19 pandemic on impulse buying based on event systems theory, which enriched the application scenario of event systems theory and extended the study of the effects of public health events to the consumer field. Second, this study extended the previous findings by further exploring the mechanisms underlying the impact of the COVID-19 pandemic on impulse buying, namely, the mediating role of the sense of control and anxiety. Third, this study introduced the specific cultural value of moderate thinking into public health and consumer research from the perspective of individual differences, providing a research basis for understanding the effects of cultural values influencing public health events. Finally, this study provides guidance on how governments and related organizations can properly guide and intervene in individuals’ awareness of the COVID-19 pandemic.

## Figures and Tables

**Table 1:** Regression analysis results.

Model:	Model 1	Model 2	Model 3	Model 4	Model 5	Model 6
Dependent Variable:	Sense of Control	Anxiety	Impulse Buying	Impulse Buying	Impulse Buying	Impulse Buying
Gender	−0.18	0.08	0.21	0.12	0.18	0.13
	(0.14)	(0.17)	(0.18)	(0.17)	(0.17)	(0.17)
Age	−0.06	−0.15	−0.35 **	−0.37 **	−0.29 *	−0.33 **
	(0.10)	(0.12)	(0.12)	(0.12)	(0.12)	(0.12)
Edu	0.04	0.05	0.02	0.04	−0.00	0.02
	(0.07)	(0.09)	(0.10)	(0.10)	(0.09)	(0.09)
COVID-19 pandemic	−1.59 ***	1.76 ***	0.62 ***	−0.13	−0.01	−0.27
	(0.13)	(0.17)	(0.18)	(0.21)	(0.21)	(0.22)
Moderate thinking	0.36) ***	−0.33 ***	−0.16	0.02	−0.04	0.03
	(0.08)	(0.08)	(0.09)	(0.09)	(0.09)	(0.09)
COVID-19 pandemic	0.41 **	−0.74 ***	−0.52 **	−0.33*	−0.26	−0.23
×Moderate thinking	(0.15)	(0.16)	(0.18)	(0.16)	(0.18)	(0.16)
Sense of control				−0.47 ***		−0.32 **
				(0.10)		(0.12)
Anxiety					0.36 ***	0.21 *
					(0.08)	(0.10)
Constant	4.86 ***	3.48 ***	4.52 ***	6.83 ***	3.27 ***	5.35 ***
	(0.51)	(0.62)	(0.68)	(0.75)	(0.69)	(0.97)
R2	0.47	0.41	0.13	0.23	0.22	0.25
VIF	1.04	1.04	1.30	1.04	1.24	1.59
N	201	201	201	201	201	201

Note: Robust standard errors in parentheses, * *p* < 0.05, ** *p* < 0.01, *** *p* < 0.001.

**Table 2:** Moderated-mediation test results.

Moderator	Coef. of the Indirect Effect	Bootstrap Std. Err.	z	*p*-Value	Normal-Based [95% Conf. Interval]		
Path a: COVID-19 pandemic→Sense of control→Impulse buying							
Low	0.01	0.08	0.07	0.942	−0.16	0.17	(P)
					−0.14	0.19	(BC)
Medium	−0.18 **	0.05	−3.46	0.001	−0.29	−0.09	(P)
					−0.30	−0.10	(BC)
High	−0.36 **	0.11	−3.41	0.001	−0.58	−0.16	(P)
					−0.62	−0.18	(BC)
Path b: COVID-19 pandemic→Anxiety→Impulse buying							
Low	0.14 *	0.07	2.02	0.044	0.03	0.29	(P)
					0.04	0.32	(BC)
Medium	−0.12 **	0.04	−3.15	0.002	−0.19	−0.05	(P)
					−0.21	−0.06	(BC)
High	−0.37 ***	0.10	−3.66	0.000	−0.58	−0.18	(P)
					−0.58	−0.18	(BC)

Note: Robust standard errors in parentheses. * *p* < 0.05, ** *p* < 0.01, *** *p* < 0.001.

## Data Availability

Due to the confidentiality of the subjects’ privacy, data can be obtained by contacting the authors.

## Appendix A. Measurement

Sense of Control (Study 2: *α* = 0.94, CR = 0.94, AVE = 0.77; Study3: *α* = 0.88, CR = 0.88, AVE = 0.60.)

1. What happens in my life is beyond my control. (R)

2. I feel helpless in dealing with the problems of life. (R)

3. I feel my life is in my control.

4. I can do just about anything I really set my mind to.

5. When I really want to do something, I will find a way to succeed at it.

Anxiety (Study 2: *α* = 0.95, CR = 0.95, AVE = 0.83; Study3: *α* = 0.94, CR = 0.94, AVE = 0.78.)

1. Tense.

2. Nervous.

3. Anxious.

4. Stressed.

Impulse Buying (Study 1: *α* = 0.93, CR = 0.93, AVE = 0.82; Study 2: *α* = 0.90, CR = 0.90, AVE = 0.75; Study 3: *α* = 0.87, CR = 0.88, AVE = 0.71.)

1. In this scenario, I will buy things I don’t need.

2. In this scenario, I will buy things I did not plan to buy.

3. In this scenario, I will buy what I don’t intend to buy.

Moderate Thinking (Study3: *α* = 0.95, CR = 0.95, AVE = 0.60.)

1. When discussing opinions, I balance conflicting views at the same time.

2. I often think about the same thing from multiple perspectives.

3. When voting, I listen to all opinions.

4. When deciding, I think about all possible outcomes.

5. I will try to find a majority opinion in a situation where there is a difference of opinion.

6. I will try to balance my own opinions with those of others.

7. I will refer to the opinions of others and adjust my original ideas.

8. I want to reach a consensus in the discussion process.

9. I will try to incorporate my own opinions into the ideas of others.

10. I generally use tactful ways to express disagreements.

11. I will try to use harmony to get a small group of people to agree with the majority.

12. I generally make decisions about opinions that take into account the harmony of the group atmosphere.

13. When making decisions, I generally adjust my delivery to maintain overall harmony.

## Appendix B. Reliability and Validity

**Table A1:** Descriptive statistics and correlations of Study 2.

Variable Name	Mean	SD	Min	Max	1	2	3
1. Sense of Control	4.59	1.61	1	7	0.88		
2. Anxiety	3.52	1.78	1	7	−0.69 ***	0.91	
4. Impulse Buying	4.17	1.58	1	7	−0.53 ***	0.53 ***	0.86

Note: *** *p* < 0.001.

**Table A2:** Descriptive statistics and correlations of Study 3.

Variable Name	Mean	SD	Min	Max	1	2	3	4
1. Sense of Control	4.67	1.26	1	7	0.77			
2. Anxiety	3.45	1.49	1	7	−0.74 ***	0.88		
3. Moderate Thinking	5.61	0.96	1.31	7	0.21 ***	−0.12 *	0.84	
4. Impulse Buying	4.02	1.35	1	7	−0.41 ***	0.42 ***	−0.07	0.77

Note: * *p* < 0.05, *** *p* < 0.001.
